# The Quantitative Imaging Network in Precision Medicine

**DOI:** 10.18383/j.tom.2016.00190

**Published:** 2016-12

**Authors:** Robert J. Nordstrom

**Affiliations:** Cancer Imaging Program, National Cancer Institute, Division of Cancer Treatment and Diagnosis, Bethesda, Maryland

**Keywords:** Quantitative Imaging Network, precision medicine, therapy response

## Abstract

Precision medicine is a healthcare model that seeks to incorporate a wealth of patient information to identify and classify disease progression and to provide tailored therapeutic solutions for individual patients. Interventions are based on knowledge of molecular and mechanistic causes, pathogenesis and pathology of disease. Individual characteristics of the patients are then used to select appropriate healthcare options. Imaging is playing an increasingly important role in identifying relevant characteristics that help to stratify patients for different interventions. However, lack of standards, limitations in image-processing interoperability, and errors in data collection can limit the applicability of imaging in clinical decision support. Quantitative imaging is the attempt to extract reliable, numerical information from images to eliminate qualitative judgments and errors for providing accurate measures of tumor response to therapy or for predicting future response. This issue of *Tomography* reports quantitative imaging developments made by several members of the National Cancer Institute Quantitative Imaging Network, a program dedicated to the promotion of quantitative imaging methods for clinical decision support.

## Introduction

The ability to make accurate measurements or predictions regarding the response of tumors to a cancer therapy early in a clinical trial can facilitate adaptive trial strategies whenever alternative therapies are available. Imaging, in its many forms, is a useful clinical method for measuring therapy response, but uncertainty in the measurements can obscure the early response trends. While patient-to-patient variability will also confound therapy response measurements, it is possible to reduce these biological effects through suitably powered clinical trials. The physical (instrumental) uncertainties caused by system errors or scanner design differences can be reduced using appropriate protocols and software corrections during data collection and image processing. These corrective actions can ideally lead to the condition where clinical imaging will provide quantitative, objective, and systematic information concerning tissue condition from digital images (1–2).

Quantitative imaging begins with an evaluation of the performance characteristics of existing imaging hardware for determining the degree of bias and variance present and how these vary over time. This is achieved through the use of phantoms, preferably traced to standards such as those from the National Institute for Standards and Technology. In this issue of *Tomography*, the Mount Sinai team in the Quantitative Imaging Network (QIN) reports on the use of phantoms for quantitative imaging research in diffusion-weighted imaging. Once the errors related to data collection are known, procedures to harmonize performance among different makes and models of imaging platforms can be studied with the goal of reducing the errors. The reduction of bias and variance from devices is a necessary strategy for quantitative imaging, but it is insufficient. Robust algorithms that are capable of extracting measurable information from the images are also needed. These algorithms must be thoroughly tested and validated in clinically relevant environments to expose them to different clinical conditions that they might encounter in future clinical trials.

If quantitative imaging is to become more than a research curiosity, it must demonstrate its usefulness in the domain of precision medicine. Here, individual characteristics of the patients are used to determine an appropriate healthcare course (3). Quantitative imaging can be an important player in the role of stratifying patients through accurate measurements of their tissue and disease characteristics to determine appropriate interventions. This is the vision and goal of the National Cancer Institute (NCI) Quantitative Imaging Network.

### Remarks

The QIN is an NCI Cancer Imaging Program and Radiation Research Program joint initiative to bring quantitative imaging methods into clinical utility, measuring response to therapy, and supporting clinical decision-making during clinical trials. This program was initiated in 2008 by the author and Dr. Laurence Clarke, both members of the Cancer Imaging Program of the NCI. The passing of Larry in April 2016 has left a void in our management team, and it will be difficult to recover much of the enthusiasm and commitment of direction that Larry provided. However, the creativity of the network members and their dedication to the advancement of quantitative imaging will continue to give impetus to the network and move it toward improved clinical decision support through quantitative imaging. This issue of *Tomography* is a tribute to Larry's vision and efforts in this area.

Each team in the network is focused on its choice of a cancer imaging problem important to the improvement of quantitative results from clinical images. In general, this involves the reduction of bias and variance in the data collection process, the identification and extraction of information from specific regions of an image or groups of images using well-validated algorithms, and the solution to problems of software interoperability and other informatics issues. Several articles in this issue of *Tomography* deal with topics in these areas. For example, a paper on the topic of data collection reproducibility from the Vanderbilt University and the University of Texas at Austin teams reports on quantitative magnetization transfer imaging in the breast at 3.0 T. These activities engage a number of different disciplines including imaging technology, oncology, radiology, informatics, and statistics; therefore, the teams must be diverse in their make-up.

Not all QIN teams are directly involved in these listed activities, however. A number of equally important projects participating in the QIN to move quantitative imaging to clinical utility include such diverse challenges as measuring the effects of reduced computed tomography (CT) dose on the ability to extract accurate quantitative information from images, transforming magnetic resonance spectroscopy as a clinically viable imaging tool, studying cost/benefit breakpoints for using quantitative methods in clinical trials, and building a streamlined informatics infrastructure in quantitative imaging. A discussion of response assessment in clinical trials and an overview of quantitative imaging methods for clinical trials are given in greater detail in the study by Yankeelov et al. (4).

The QIN is organized to promote consensus discussions and facilitate the sharing of data and tools. Teams enter the network through the standard National Institutes of Health process of peer review, writing an application to the specific program announcement (5). Once selected for admission into the network, each team is responsible for successful completion of its research plan. In addition, there are network functions required of them. An Executive Committee, consisting of the principal investigators from each of the teams, meets by teleconference once every month to discuss network directions such as connecting with clinical trial activities in the National Clinical Trials Network, interactions with professional societies, annual meeting events, and much more. Working groups are the forums where the network teams interact on specific topics. Each research team is expected to provide members to each of the different working groups. Therefore, the working groups each become a microcosm of the entire network focused on specific technical and clinical issues. A diagram of the QIN network organization has appeared elsewhere (1). The current working groups include:

- Clinical Trail Design and Development.
- Bioinformatics/IT and Data Sharing.
- Data Acquisition.
- Image Analysis and Performance Metrics.
- Positon emission tomography (PET)/CT Subgroup.
- MRI Subgroup.

Several papers in this issue have originated from QIN working groups rather than from the research teams, showing the dedicated commitment of the QIN teams to the working group process. The Clinical Trial Design and Development Working Group, for example, is reporting the results of clinical trial accrual statistics for trials involving quantitative imaging studies. Looking at the correction of errors during data collection, the Data Acquisition Working Group discusses its efforts in validating gradient nonlinearly corrections in diffusion-weighted magnetic resonance imaging (MRI). The PET/CT Subgroup of the Image Analysis and Performance Metrics Working Group is reporting on a multi-institutional study of feature robustness in radiomics of lung cancer.

Radiomics is a rapidly growing area of quantitative imaging, and several teams within QIN are emphasizing this approach for deriving the quantitative features in images that will correlate with the outcome or provide reliable measures of tumor response to therapy. In radiomics, a large set of advanced imaging features is used to extract predictive phenotypic tumor information (6). From this very large number of initial features, the goal is to reduce the feature set to a stable subset of reasonable size (eg, 7) that continues to hold predictive potential (eg, pathological response). The Stanford University team has an article in this issue that discusses rapid radiomic feature extraction in non-small cell lung cancer.

Not all feature extractions are connected with radiomics, however. Texture feature extraction in dynamic contrast-enhanced MRI images during early-stage breast cancer therapy is reported by the Oregon Health and Science University. Other tumor sites that are reported on in this issue include bladder, lung, and brain using several quantitative imaging methods and algorithms. In addition, another of the QIN teams from the University of Michigan is reporting on temporal feature extraction from dynamic contrast-enhanced MRI to identify poorly perfused tumor subvolumes in head and neck cancers. In another article, the same team is also reporting on quantitative evaluation of apparent diffusion coefficient measurements in head and neck cancer.

### Translation into Clinical Workflow

As the research from each team in the QIN moves from development and testing into clinical validation, the research activities are drawn away from solving technical issues to focus on questions of quality assurance, standardization, and translational methods. With few exceptions, these tasks are foreign to most academic researchers; yet, this translation of effort is a necessary step if quantitative imaging is to participate in the precision medicine environment. The coordination of imaging with clinical workflow can be complex, and the details of how advanced imaging will be incorporated into clinical trials will vary across institutions. This difficulty grows when multisite trials are considered. One of the articles in this issue is addressing the placement of quantitative imaging in clinical workflow.

The QIN is now in a position to begin addressing the tasks that will overcome the barriers to clinical translation. One important activity here is the initiation and conduct of algorithm challenges. A number of algorithms with similar functions are tested against a common data set to compare the results. In the network environment, it is not a matter of either winning or losing that is at stake in these challenges. Instead, it is the genuine desire to make improvements in the algorithm through tool-sharing and consensus-building within the network that motivates the challenges. The process adapted by the network for planning, conducting, and reporting software challenges is the subject of an article submitted by the Challenge Taskforce responsible for creating the guidelines.

While challenges can shed light on which tools are advanced enough to be considered for more extensive clinical exposure, these do not accelerate the translation of tools into clinical workflow. This can only be done by understanding the pathways through the translational landscape and selecting one that appears to be optimum. Of course, all paths lead eventually to discussions with the Food and Drug Administration, but before that, there must be an extensive clinical validation of tool performance through contacts with organizations such as the National Clinical Trials Network, where tools can be placed in ongoing clinical trials on a test basis. This is the current goal of the QIN.

## Conclusions

The articles in this issue of *Tomography* demonstrate a number of efforts in QIN, leading to the advanced development of quantitative tools for clinical decision support and show the results of collaboration in the network through working group involvement. There are numerous activities in the network that are not being reported here, however. The network recognizes that there is a large body of work that must be done after the development phase. This is the challenge of deployment into clinical trials. The NCI is very interested in accelerating this transition within QIN and is working to emphasize the importance of this evolution. Only then can quantitative imaging be a partner in precision medicine.
